# Low-Cost iPhone-Assisted Processing to Obtain Radiotherapy Bolus Using Optical Surface Reconstruction and 3D-Printing

**DOI:** 10.1038/s41598-020-64967-5

**Published:** 2020-05-15

**Authors:** Dehua Kang, Bin Wang, Yinglin Peng, Xiaowei Liu, Xiaowu Deng

**Affiliations:** 1 School of Physics, Sun Yat-sen University, No. 135, Xingang Xi Road, Guangzhou, 510275 China; 2 Department of Radiation Oncology, State Key Laboratory of Oncology in South China, Collaborative Innovation Center for Cancer Medicine, Sun Yat-sen University Cancer Center, Guangzhou, 510060 China

**Keywords:** Skin cancer, Skin cancer

## Abstract

Patient specific boluses can increase the skin dose distribution better for treating tumors located just beneath the skin with high-energy radiation than a flat bolus. We introduce a low-cost, 3D-printed, patient-specific bolus made of commonly available materials and easily produced using the “structure from motion” and a simple desktop 3D printing technique. Nine pictures were acquired with an iPhone camera around a head phantom. The 3D surface of the phantom was generated using these pictures and the “structure from motion” algorithm, with a scale factor calculated by a sphere fitting algorithm. A bolus for the requested position and shape based on the above generated surface was 3D-printed using ABS material. Two intensity modulated radiation therapy plans were designed to simulate clinical treatment for a tumor located under the skin surface with a flat bolus and a printed bolus, respectively. The planned parameters of dose volume histogram, conformity index (CI) and homogeneity index (HI) were compared. The printed bolus plan gave a dose coverage to the tumor with a CI of 0.817 compared to the CI of 0.697 for the plan with flat bolus. The HIs of the plan with printed bolus and flat bolus were 0.910 and 0.887, respectively.

## Introduction

Bolus is commonly used in radiotherapy for treating tumor near the skin. It offers a water equivalent material to provide sufficient coverage of the tumor and a homogenous dose distribution^1^. However, commercially available flat bolus fabricated of sheet gels cannot be easily applied to some irregular skin surfaces like those of the nose, ears, eyes, and scalp. The most important issue with these flat boluses is the air gap between the bolus and the skin, especially when using a large beam incident angle in an intensity-modulated-radiotherapy plan, which can decrease the surface dose^2,3^. The idea of a customized bolus was proposed to increase the irregular surface dose and enhance the homogeneity of dose distribution to the tumor in the buildup region. Three-dimensional (3D) printed boluses are being increasingly applied in modern radiotherapy in recent years^4–6^. Thus, patient-specific boluses can be printed using the patient’s surface data from imaging or other 3D surface scanning devices. A common way to design 3D-printed, patient-specific boluses is based on the patient’s computed tomography (CT) image data, which requires the application of two CT scans to the patient—the first scan is to acquire image data for reconstructing the surface shape for printing and the second scan is conducted with the 3D-printed bolus on for treatment planning. The patient would therefore receive an extra X-ray irradiation. Some other designs based on optical scanning of the patient have also been proposed^7,8^. The currently used methods are expensive or require complicated processing. In this study, we proposed a new method to acquire surface images by using a cell phone camera and successfully used the structure from motion (SFM) method to reconstruct the 3D surface, along with a unique scale calibration procedure, for printing patient-specific boluses.

## Results

**Figure 1:**
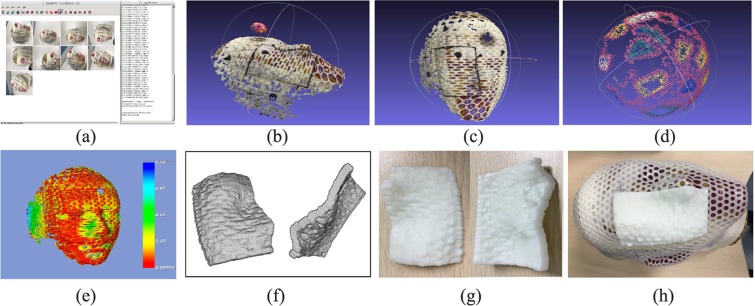
Procedure of bolus reconstruction using the SFM method. (**a**) The acquired pictures were imported into SFM workspace and 3D reconstruction was run. (**b**) The surface of the head phantom and the sphere calibration model. (**c**) The surface of head phantom with a bolus region marker line. (**d**) The surface of the sphere calibration model. (**e**) The registration deviation between the two surfaces from the SFM and Marching Cube from CT images. (**f**) Bolus viewed in the STL format file. (**g**) The bolus printed using ABS material. (**h**) The bolus was put in the right place on the head phantom surface.

The Fig. 1 showed the all steps needed in the patient specific bolus making procedure. The reconstructed surface from images was rescaled using calibration model method. The rescaled surface showed the good conformity with CT surface of the phantom checked using registration between them. A conformal bolus was 3D printed using acrylonitrile butadiene styrene (ABS) material from the ply format file.

The CI and HI of the radiation treatment plans with patient-specific printed and standard flat bolus were 0.817 and 0.910 (printed) vs 0.697 and 0.887 (flat), respectively. The prescription dose coverage for PTV in the plan with printed bolus were much better than that in the plan with flat bolus. The V95% (percentage volume received at least 95% of prescription dose) and D95% (dose covered 95% of the volume) in the PTV were 95.65% and 47.96 Gy (printed) vs 88.39% and 46.11 Gy (flat), while the dose value in every OAR were very similar for the two plans, respectively. (Tables 1 and 2).

**Table 1:** Comparison of HI, CI,V_95%_ and D_95%_ values of PTV between the printed bolus and flat bolus plan.

	D_min_(GY)	D_mean_(GY)	D_max_(GY)	CI	HI	V_95_(%)	D_95_(Gy)
Printed bolus	29.99	50.65	54.19	0.817	0.910	95.65	47.96
Flat bolus	28.73	49.61	53.31	0.697	0.887	88.39	46.11

**Table 2:** Comparison of parameters of OARs between the printed bolus and flat bolus plan.

Structures		Printed bolus (Gy)	Flat bolus (Gy)
Left Lens	D_mean_	0.91	0.74
	D_max_	2.09	1.26
Right Lens	D_mean_	1.84	1.43
	D_max_	3.64	2.61
Left Eye	D_mean_	0.74	0.73
	D_max_	2.92	2.74
Right Eye	D_mean_	1.69	1.60
	D_max_	8.42	8.27
Left Middle Ear	D_mean_	3.85	3.03
	D_max_	7.77	6.60
Right Middle Ear	D_mean_	6.38	5.63
	D_max_	10.46	9.32
Left Parotid	D_mean_	3.73	3.58
	D_max_	5.89	6.07
Right Parotid	D_mean_	4.20	4.14
	D_max_	8.43	7.47

**Figure 2:**
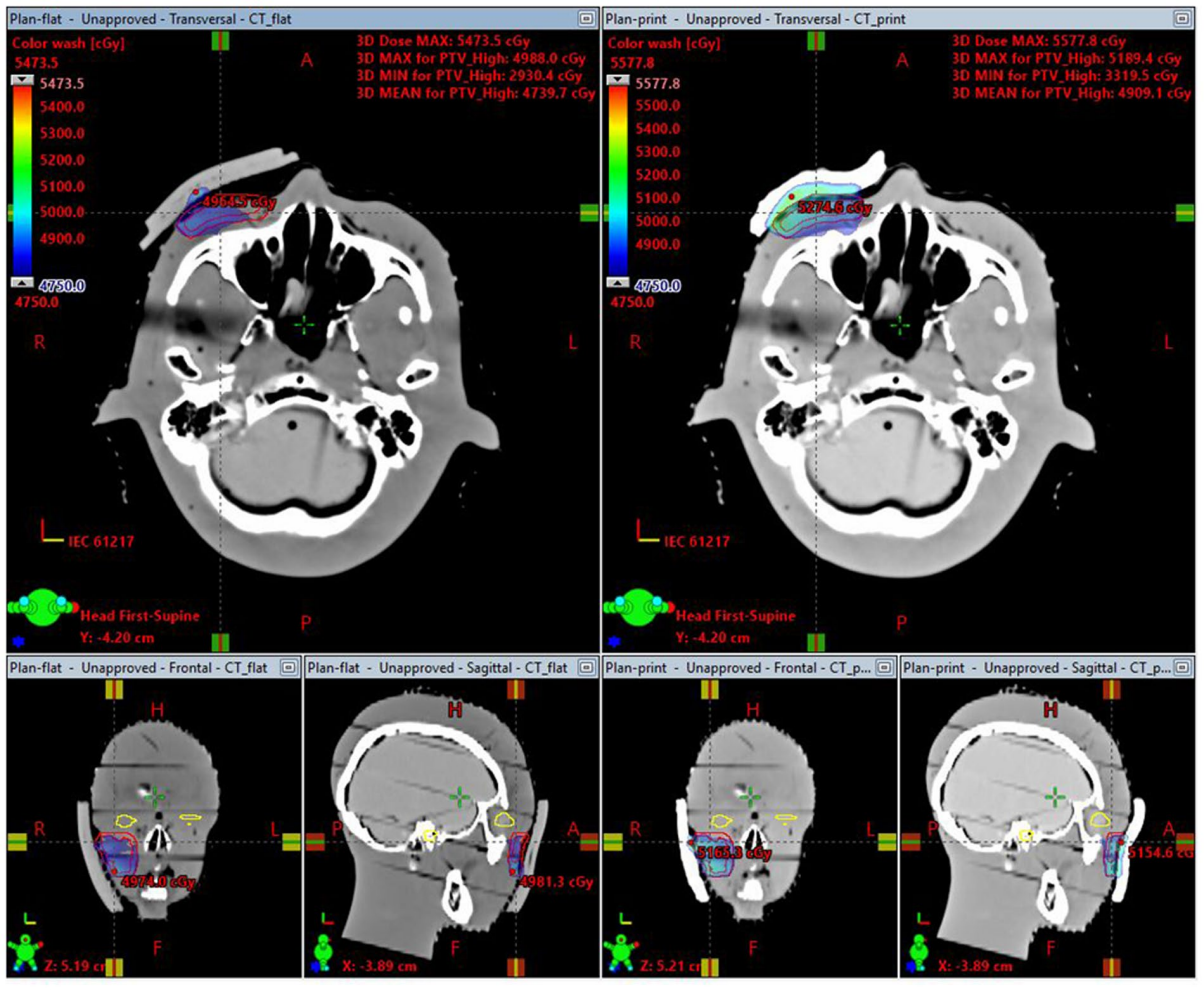
The left panel shows the dose distribution of the 5-beam IMRT plan without a piece of flat bolus. The right panel shows the dose distribution of the 5-beam IMRT plan with printed bolus. The minimum dose color wash was 4750 cGy. The bottom are the sagittal and coronal views according to the corresponding plans, respectively.

**Figure 3:**
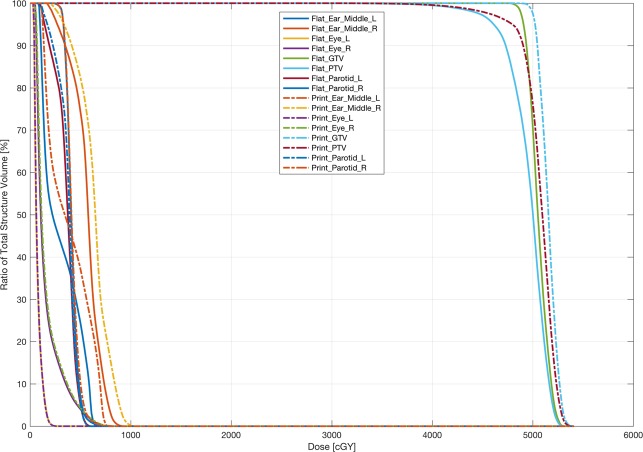
DVH of the two plans with printed bolus and a piece of flat bolus, respectively.

The dose distribution of typical slices and the comparison of DVHs for the two plans are shown in Figs. 2 and 3. The results demonstrated that the dose coverage and conformity of the plan with printed bolus was superior to that with flat bolus, with a higher dose coverage in the superficial PTV area.

## Discussion

3D printing technologies are becoming popular to build complex volumetric objects like shaped boluses and compensators to be applied in radiotherapy to improve the percentage depth dose of superficial or build up area and to compensate for the irregular surface of patient skin. The design of these types of boluses is usually based on CT images and proposed in the TPS^9,10^. In these process, the patient has to undergo CT scanning twice, even for the patients with skin cancer whose tumor area can be easily visually defined, once for bolus shape designing and the other time for dose calculation with the bolus on. Hence, the patient needs to undergo an extra CT scanning.

Our phantom simulation shown that the plan with printed 3D bolus gave a better dose distribution than the one with a standard flat bolus, having better dose coverage to the PTV and dose conformity. The V95% for the PTV were 95.65% (3D-printed bolus) vs 88.39% (flat bolus). The CI and HI of the plan with 3D-printed bolus raised to 0.817 and 0.910 from 0.697 and 0.887 of that with a flat bolus, respectively. The superiority came from the better fit of the printed bolus to the irregular surface that the flat bolus was not able to conformally cling on to the skin sags and crests. The simulation results demonstrated that a big air gap was existed between the flat bolus and the skin decreased the dose coverage to the PTV. Richard *et al*.^10^ reported their comparing plan study of using 3D-printed bolus vs conventional manually created bolus for skin cancer treatment, the V85% of the CTV was on average 97% (3D-print) vs 88% (conventional). The result was similar with our study but their 3D-print was based on the CT data set.

There were a few reports using other optical capturing method to acquire the surface data for 3D bolus printing, Sharma, A.^7^ *et al*. reported a similar procedure of using a gantry mounted infrared camera to scan the patient and reconstruct the 3D surface with renderings of meshes. The processing is quite complicated and needs a set of special made device, including expensive inferred camera and an iso-centric rotating gantry. In our study, we proposed a new process using a low-cost iPhone camera to acquire images of the patient body and used the SFM method to reconstruct the 3D surface for patient-specific bolus printing. Image acquisition procedure is simple but the whole target object needs to be included in the picture view. It means that every image must have an overlapping part with the neighboring one, and the surface to be reconstructed should be covered by all pictures around the object. This process reduces the patient’s visits and reduces the number of CT scanning for the patient. The materials (ABS) used for bolus printing are easily available, and the cost of the 3D printing is less, thus making the process economically adequate for wide applications in developing nations.

The SFM, which uses the scale invariant feature transform algorithm to extract the features in images, is an effective method to reconstruct 3D facial shape. However, the performance of SFM might degrade when tracking errors caused by self-occlusion or image noise exist in a dark environment^11,12^. In addition, the SFM method does not deal with space scaling so that a geometric scale has to be define for the reconstruction. Usually the scaling of the SFM is to manually measure an absolute distance in the 3D scene and then use it to scale the reconstruction for consisting its physical dimensions^13,14^. Another trend to estimate the scale for SFM is using fusing image measurement with other sensors, such as inertial measurement unit^15^, or the global positioning system^16^. These methods can be quite difficult to perform and are better suited to large-scale reconstructions for which the measurement error can be negligible compared to the distance being measured. Although it has never been reported to apply this method for shaping individual bolus or test the reconstruction accuracy in a relative dark environment like in a radiation treatment room, our study demonstrated that the SFM reconstruction works well enough for printing patient specific bolus, by capturing 9 overlapping sequenced pictures with an iPhone camera in the usually lighted treatment room and calibrating the scale with our unique designed calibration procedure.

For scale the reconstructed structure, a sphere model with textures of known geometry was used for scale calibration, which ensured accurate 3D reconstruction to design the bolus conformally onto the patient’s irregular body. The radius of the sphere model was set to 15 mm because a larger sphere would block the head phantom, while a smaller sphere would lead to low accuracy in the reconstruction result. The 3D sphere fitting algorithm to fit the sphere surface is a robust and accurate method. The ratio between the fitting radius and the known radius was used as the scaling factor for the reconstructed structure. In the 3D surface scene, it is more difficult and complicated to measure the distance between two points on the irregular surface, but the radius of a sphere can be easily determined using the least square fitting method.

**Figure 4:**
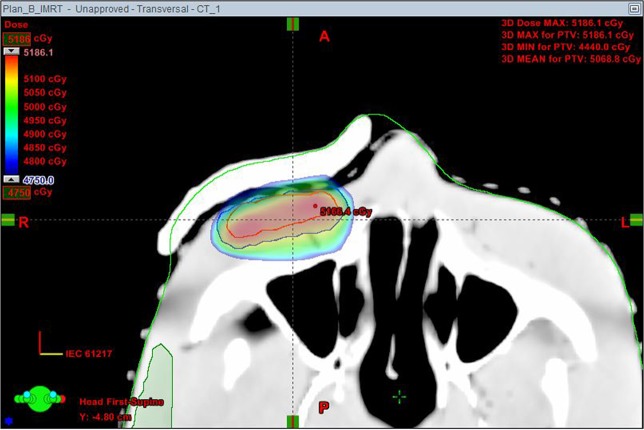
Details of the gap between the printed bolus and the head phantom surface.

In the clinic application simulation of the printed bolus, there is still a small gap existed between the printed bolus and the head phantom surface (Fig. 4). This was caused by the immobilization with the thermoplastic mask and could be improved by setting the bolus onto the patient skin directly under the thermoplastic mask in future practice.

## Conclusion

A low-cost and easy method using an iPhone to produce a patient-specific bolus with 3D-printing for application in radiation therapy is established. This process is suitable for the skin cancer, as the PTV can be defined based upon the appearance. The methods in this paper can make it possible to scan the planning CT with a printed bolus together. The proper geometry and density can be recognized by the TPS thus an accurate dose calculation will be achieved. The simulation plan shows that the printed bolus was satisfactory for application to improve the dose coverage and conformity in IMRT treatment for a superficial target in the head and neck areas.

### Abbreviations

ABS: acrylonitrile butadiene styrene; IMRT: intensity-modulated radiation therapy; TPS: treatment planning system; GTV: gloss tumor volume; PTV: planning target volume; OARs: organs at risk; CI: conformity index; CT: computed tomography**;** HI: homogeneity index; DVH: dose volume histogram;V95%: the percent volume that received at least 95% of the prescription dose; D95%: the percent of the prescription dose covering 95% of the volume; SFM: structure from motion; ICP: Iterative Closest Point; VTK: The Visualization Toolkit.

## Methods

An anthropomorphic head RANDO phantom (Alderson Research Laboratories, Stanford, USA) with a textured sphere model attached on the surface was used for reconstruction and imaging using an iPhone camera. The SFM^17,18^ method was used to convert the images of the target to reconstruct the 3D surface structure. A 3D sphere fitting algorithm was used to calibrate the scale of the reconstruction. The CT image set of the head phantom was used to verify the scale using iterative closest point registration. MeshLab^19^ software was used to extract the shape of the bolus from the reconstructed surface; C++ algorithm using the VTK^20^ library was applied to generate the proper bolus thickness, output the STL format file to a 3D-printer, and process bolus printing with acrylonitrile butadiene styrene (ABS) material. The printed bolus was then attached to the suitable position of the head phantom and rescanned with the CT simulator. The CT images with bolus were input to the treatment planning system TPS (Eclipse V15, Varian Medical System, Palo Alto, CA. USA) to simulate a clinical plan using the bolus and to evaluate dose distribution.

### Phantom surface reconstruction from SFM

The SFM method, described by Wu *et al*.^17^, was used to generate the 3D surface in our study, which was designed to process and reconstruct the 3D structure from a set of 2D images. A portion of these 2D images must be overlapped with neighboring pictures of the same target, so that all features of the target could be extracted and correspondingly matched one by one. The camera angle and location were calculated using the bundle adjustment algorithm^18^ from two-view matches. The information in these images was then projected into 3D space to reconstruct the 3D surface structure using the camera’s pose and location which were acquired from above calculation. Because of that the SFM reconstruction is based on relative space relationship, it needs a scale to restore the real size of the reconstructed object. In our study, a sphere calibration model was introduced to scale the reconstructed 3D structure using the sphere fitting algorithm to obtain the sphere radius and calculate the radius ratio of the reconstructed sphere and its physical size.

#### Phantom image acquisition

The head phantom was used to simulate a patient with a tumor under the orbital region. The head phantom was immobilized using a thermal plastic mask for clinical positioning. The bolus region was marked using a marking pen. The sphere calibration model was positioned and stuck on to the head phantom near the orbital region where the bolus was required. Nine overlapping images were acquired around the head phantom using an iPhone (iPhone 6 plus, Apple Inc. Cupertino, CA, USA) camera at a distance of approximately 40 cm to obtain every image view including the whole phantom and the sphere calibration model. The images were sequenced, and the camera was positioned in the periphery to ensure that every part of the phantom surface was imaged.

#### Image processing and scaling

The nine overlapping images were imported into the VisualSFM^21^ toolkit for feature extraction, matching, and surface reconstruction (Fig. 1a,b). Bundle adjustment and dense reconstruction functions are included in the VisualSFM toolkit, too. The reconstructed 3D surface was then exported as a ply file format. MeshLab was used to remove the noise in the reconstructed 3D surface, separate the sphere calibration model, and cut the bolus region from the reconstructed surface (Fig. 1c,d). The data of the sphere calibration model and bolus were exported as a ply file to be used in the next part of the experiments. An in-house program written with C++ and VTK library was used to develop the fitting algorithm to determine the scale factor.

Considering point ${p}_{i}\,$on the surface of a sphere, the following equation can be satisfied:

$${\Vert {p}_{i}(x,y,z)-O({x}_{0},{y}_{0},{z}_{0})\Vert }^{2}={r}^{2}$$

here $O({x}_{0},{y}_{0},{z}_{0})$ is the origin of the sphere, and $r$ is the radius of the sphere. According to the principle of least square method, the error equation is defined as

$${\epsilon }=\mathop{\sum }\limits_{i=1}^{N}{\Vert {r}^{2}-{({x}_{i}-{x}_{0})}^{2}-{({y}_{i}-{y}_{0})}^{2}-{({z}_{i}-{z}_{0})}^{2}\Vert }^{2}$$

The coordinates of the origin $O({x}_{0},{y}_{0},{z}_{0})$ and the radius $r$ can be obtained by minimizing the $\epsilon$ value. If the real radius of the sphere calibration model is given by $R\,(15\,mm)$, the scale factor can be described by

$$s=R/r$$

This scale factor was used to rescale the 3D bolus. The VTK library was used to process the head phantom surface, and the thickness of the bolus in the Z direction was increased using transform filter and append poly data algorithm. Finally, the processed data were exported in the STL format to print the bolus using ABS materials as demonstrated by Ricotti *et al*.^22^ and Burleson *et al*.^23^.

### Verification of the phantom surface reconstruction

The phantom surface reconstructed from the CT dataset using the marching cube algorithm was used as the ground truth standard to verify the accuracy of the 3D structure reconstructed from the SFM method. Iterative Closest Point^24^ (ICP) registration was used in VTK to register the two surfaces, where the average space deviation was calculated to be $1.7{\rm{mm}}\pm 1.1{\rm{mm}}$ for all registered points (494367 in total) on the surface and a rotation error less than 0.5° was obtained, when 150 iterations were calculated (Fig. 1e).

### Phantom plan simulation

The RANDO phantom, was scanned using the CT simulator (SOMATOM Sensation 16, Siemens Healthcare GmbH Germany) with a standard flat bolus and the printed bolus fixed on top of it, respectively, Both CT image data sets were then imported into the radiation TPS, to generate a 5-beam IMRT plan using the same dose constraints (Table 3) and same beam angles (270°, 300°, 330°, 0°, 30°). The Analytical Anisotropic Algorithm (AAA) was used to calculate the dose distribution in this study. A GTV was defined just under the skin surface, and the PTV was created by expanding a 3-mm margin to the GTV (in clinical practice, the bolus area which needed for skin cancer can be visually defined by the radiation oncologist on the cancer appearance). A marker line which enclose the whole bolus area on the skin surface or the mask can be drawn using a marker pen by the doctor (Fig. 1(b,c)). A dose of 50 Gy/25 F was prescribed for the PTV. Results of the two plans were evaluated and compared, including the minimal and maximum dose (D_min_ and D_max_) and percentage volume covered by 95% of prescribed dose (V_95%_) of the PTV, the mean and maximum dose of the OARs. Paddic’s conformity index (CI)^25^ and Oliver’s homogeneity index (HI)^26^ were used to evaluate the difference between the two plans as well.

$$CI=\frac{{(T{V}_{PIV})}^{2}}{TV\times PIV}$$

$$HI={D}_{95 \% }/{D}_{5 \% }$$

**Table 3:** The dose constraints for the two IMRT plans.

Structure	Dose-Volume constraints
PTV	D_max_ ≤ 110%
	V95% > 95%
	47.5 Gy ≤ *D*_*mean*_ ≤ 52.5 Gy
Left Lens	D_max_ ≤ 5 Gy
Right Lens	D_max_ ≤ 8 Gy
Eye unilateral	D_max_ ≤ 40 Gy
Parotid unilateral	D_max_ ≤ 45 Gy
	V_18Gy_ ≤ 33%
	D_mean_ ≤ 5Gy
Ear Middle unilateral	D_max_ ≤ 45 Gy

PTV = Planning target volume, V_95%_ = volume receiving 95% of the prescription dose, D_95%_ = dose received by 95% PTV volume, V_d_ = the volume of specific organ which receiving a dose of d Gy.

## Data Availability

The datasets are backed up on the Research Data Deposit (RDD, https://www.researchdata.org.cn, approval number: RDDB2019000707) and are available on reasonable request.
